# Supplementary material for: Identification and Confirmation of Virulence Factor Production from Fusarium avenaceum, a Causal Agent of Root Rot in Pulses
Source: J Fungi (Basel). 2024 Nov 26;10(12):821. doi: 10.3390/jof10120821 (PMC11677700; doi:10.3390/jof10120821)
Supplement: Supplementary file 1 [file jof-10-00821-s001.zip › jof-3318499-supplementary.pdf]

# Supplemental Information for:

# Identification and confirmation of virulence factor production from *Fusarium avenaceum*, a casual agent of root rot in pulses

**Thomas E. Witte[1], Anne Hermans[1], Amanda Sproule[1], Carmen Hicks[1], Tala Talhouni[1], Danielle Schneiderman[1], Linda J. Harris[1], Anas Eranthodi[2], Nora A. Foroud[2], Syama Chatterton[2], and David P. Overy[1*]**

[1] Ottawa Research & Development Centre, Agriculture & Agri-Food Canada, 960 Carling Ave., Ottawa, ON, K1A 0C6, Canada; tom.witte@agr.gc.ca (T.E.W.); anne.hermans@agr.gc.ca (A.H.); amanda.sproule@agr.gc.ca (A.S.); carmen.hicks@agr.gc.ca (C.H.); tala.talhouni@agr.gc.ca (T.T.); danielle.schneiderman@agr.gc.ca (D.S.); linda.harris@agr.gc.ca (L.H.); david.overy@agr.gc.ca (D.P.O.)

[2] Lethbridge Research & Development Centre, Agriculture and Agri-Food Canada, 5403–1st Avenue South, Lethbridge, AB, T1J 4B1, Canada; anas.eranthrodi@agr.gc.ca (A.E.); nora.foroud@agr.gc.ca (N.A.S.); syama.chatterton@agr.gc.ca (S.C.)

* Correspondence: david.overy@agr.gc.ca

**Table S1.** Canadian Collection of Fungal Cultures (CCFC) accession number, strain name, isolation year and province of origin of all *Fusarium avenaceum* isolates profiled in this study.

| CCFC Accession ID | Strain name | Year | Province of origin |
|---|---|---|---|
| DAOMC242072 | FaLH01 | 2010 | Saskatchewan |
| DAOMC242074 | FaLH02 | 2010 | Alberta |
| DAOMC242879 | FaLH03 | 2011 | New Brunswick |
| DAOMC242079 | FaLH05 | 2010 | Quebec |
| DAOMC242084 | FaLH10 | 2011 | Quebec |
| DAOMC242112 | FaLH15 | 2011 | New Brunswick |
| DAOMC242115 | FaLH18 | 2011 | New Brunswick |
| DAOMC242117 | FaLH20 | 2011 | New Brunswick |
| DAOMC242119 | FaLH22 | 2011 | Nova Scotia |
| DAOMC242121 | FaLH24 | 2011 | Nova Scotia |
| DAOMC242880 | FaLH27 | 2011 | Nova Scotia |
| DAOMC251794 | FaLH29 | 2011 | New Brunswick |
| DAOMC242379 | FaLH32 | 2011 | New Brunswick |
| DAOMC242381 | FaLH36 | 2011 | Ontario |
| DAOMC251795 | FaLH37 | 2011 | Ontario |
| DAOMC251796 | FaLH38 | 2011 | Ontario |
| DAOMC251797 | FaLH39 | 2011 | Ontario |
| NA | *Fa*LH27Δ*esyn1_2* | NA | NA |
| NA | *Fa*LH27Δ*esyn1_8* | NA | NA |

**Table S2.** Assembly statistics.

| Assembly | FaLH01 | FaLH05 | FaLH10 | FaLH15 | FaLH18 | FaLH20 | FaLH22 | FaLH24 | FaLH29 | FaLH32 | FaLH36 | FaLH37 | FaLH38 | FaLH39 |
|---|---|---|---|---|---|---|---|---|---|---|---|---|---|---|
| Total length (Mb) | 42.41 | 42.37 | 42.89 | 42.09 | 42.03 | 42.67 | 42.03 | 41.92 | 42.65 | 42.25 | 43.67 | 43.30 | 42.02 | 42.13 |
| # scaffolds | 51 | 76 | 61 | 66 | 119 | 75 | 102 | 58 | 108 | 105 | 189 | 55 | 61 | 72 |
| GC (%) | 47.9 | 47.9 | 47.9 | 47.9 | 48.1 | 47.9 | 48.0 | 48.0 | 48.0 | 48.1 | 47.9 | 47.9 | 48.0 | 48.0 |
| N50 scaffold length (Mb) | 1.52 | 1.22 | 1.68 | 1.84 | 1.56 | 1.38 | 1.43 | 1.69 | 1.22 | 1.21 | 0.93 | 1.78 | 1.51 | 1.13 |
| N75 scaffold length (Mb) | 1.04 | 0.69 | 1.25 | 0.84 | 0.86 | 0.85 | 1.09 | 1.12 | 0.76 | 0.77 | 0.41 | 0.92 | 0.93 | 0.78 |
| L50 scaffold count | 12 | 12 | 9 | 8 | 10 | 12 | 9 | 9 | 13 | 12 | 18 | 9 | 11 | 13 |
| L75 scaffold count | 20 | 24 | 16 | 17 | 20 | 22 | 18 | 17 | 24 | 22 | 36 | 17 | 20 | 24 |
| # N's per 100 kbp | 5.02 | 3.3 | 5.5 | 2.16 | 1.76 | 1.5 | 1.21 | 2.15 | 2.93 | 2.6 | 4.63 | 2.32 | 2.9 | 2.16 |
| Average k-mer coverage * | 63.5 | 15.7 | 83.1 | 30.24 | 23 | 25.4 | 36.4 | 22.7 | 15 | 13.6 | 10.7 | 21.2 | 20.4 | 21.8 |
| BUSCO completeness (%)** | 99.8 | 99.8 | 99.8 | 99.8 | 99.9 | 99.8 | 99.8 | 99.8 | 99.8 | 99.8 | 99.8 | 99.8 | 99.7 | 99.8 |

* calculated at highest k-mer used by SPAdes during assembly (k=99).

** database = Hypocreales_ODB10

**Table S3**. Mass features annotations, predicted chemical formulas, chromatographic retention times (RT), observed protonated ([M+H]+) mass to charge ratio (*m/z*) and mass accuracy compared to calculated exact masses of annotated molecules. Annotation levels refer to methods, where 1 = *m/z* match (<5 ppm), 2 = top MS-FINDER filtered match to reference structure library, 3 = MS-DIAL match of mass feature $MS^2$ to MassBank $MS^2$ reference library, 4 = match to chemical standard.

| Compound name | Chemical formula | RT | Precursor *m/z* [M+H]+ | accuracy (ppm) | Annotation level |
|---|---|---|---|---|---|
| AOD | C20H43NO | 6.03 | 314.34134 | 1.28 | 2 |
| Acuminatopyrone | C11H11NO3 | 2.5 | 206.08154 | 1.65 | 1 |
| Antibiotic Y | C15H10O8 | 4.48 | 319.04474 | 0.33 | 2 |
| Aurofusarin | C30H18O12 | 4.95 | 571.08704 | 0.11 | 3 |
| Chlamydospordiol | C11H16O5 | 3.06 | 229.10764 | 0.17 | 1 |
| Chlamydosporols | C11H14O5 | 3.32, 3.41 | 227.09192 | 2.29 | 4 |
| Chrysogine | C10H10N2O2 | 3.09 | 191.08173 | 0.226 | 1 |
| Enniatin A | C36H63N3O9 | 6.61 | 682.46484 | 1.67 | 4 |
| Enniatin A1 | C35H61N3O9 | 6.39 | 668.45044 | 2.75 | 4 |
| Enniatin B | C33H57N3O9 | 5.99 | 640.41949 | 3.45 | 4 |
| Enniatin B1 | C34H59N3O9 | 6.17 | 654.43494 | 3.12 | 4 |
| FDDP-shunt | C28H42O4 | 5.93 | 443.3164 | 1.93 | 1 |
| FDDP-D | C29H42O7 | 5.71 | 503.30157 | 2.46 | 4 |
| FDDP-E | C29H42O6 | 6.15 | 487.30649 | 2.21 | 4 |
| Fusahexin | C30H50N6O7 | 4.71 | 607.3811 | 0.45 | 1 |
| Fusahexins (hydrolyzed) | C30H52N6O8 | 4.26, 4.43 | 625.39368 | 2.78 | 1 |
| Fusaoctaxin A | C36H68N8O10 | 3.95 | 773.51324 | 0.16 | 1 |
| Fusaoctaxin analog 2 | C36H68N8O9 | 3.93 | 757.51762 | 1.13 | 1 |
| Fusaoctaxin analog 3 | C35H67N10O9 | 3.93 | 771.5085 | 1.1 | 1 |
| Fusaoctaxin analog 4 | C36H69N10O10 | 3.98 | 801.51561 | 3.57 | 1 |
| Fusapentaxin A | C21H39N5O7 | 3.32 | 474.29016 | 2.07 | 1 |
| Fusarin A | C23H29NO6 | 4.96 | 416.206545 | 0.54 | 2 |
| Fusarin C | C23H29NO7 | 4.61 | 432.20166 | 0.043 | 2 |
| Fusarin PM | C22H27NO7 | 4.02 | 418.18588 | 0.36 | 2 |
| Fusaristatin A | C36H58N4O7 | 5.78 | 659.43787 | 0.066 | 2 |
| Fusatrixin A | C15H31N3O4 | 3.57 | 318.23821 | 0.86 | 1 |
| Fuscofusarin | C30H20O11 | 5.38 | 557.10803 | 0.35 | 2 |
| JM-47 | C21H36N4O6 | 3.5 | 439.25485 | 0.596 | 2 |
| N-desmethyl sambutoxin | C27H7NO4 | 5.88 | 440.27936 | 0.4 | 2 |
| Rubrofusarin | C15H12O5 | 5.33 | 273.07544 | 1.14 | 3 |
| Sambutoxin | C28H39NO4 | 6.12 | 454.29507 | 0.25 | 2 |

A)

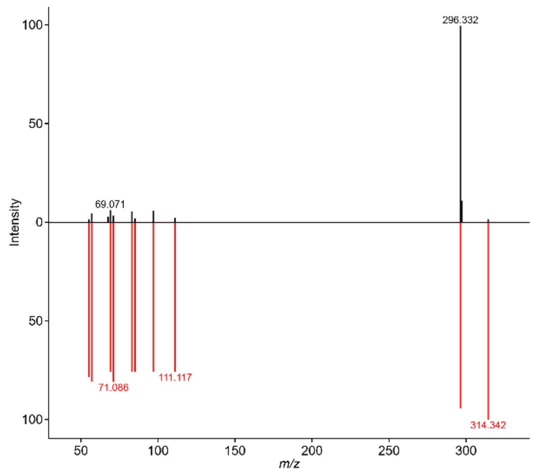


B)

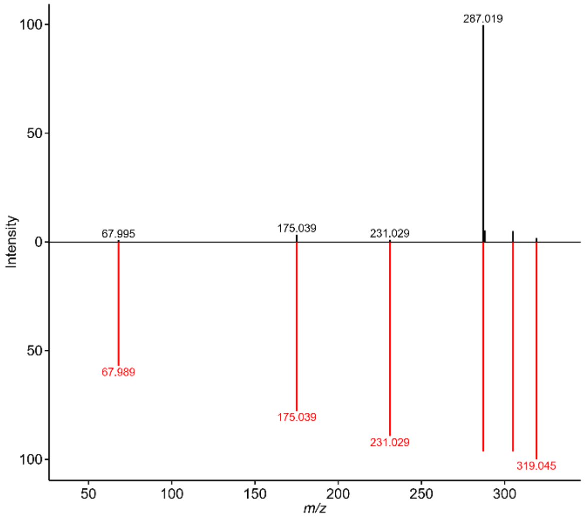


C)

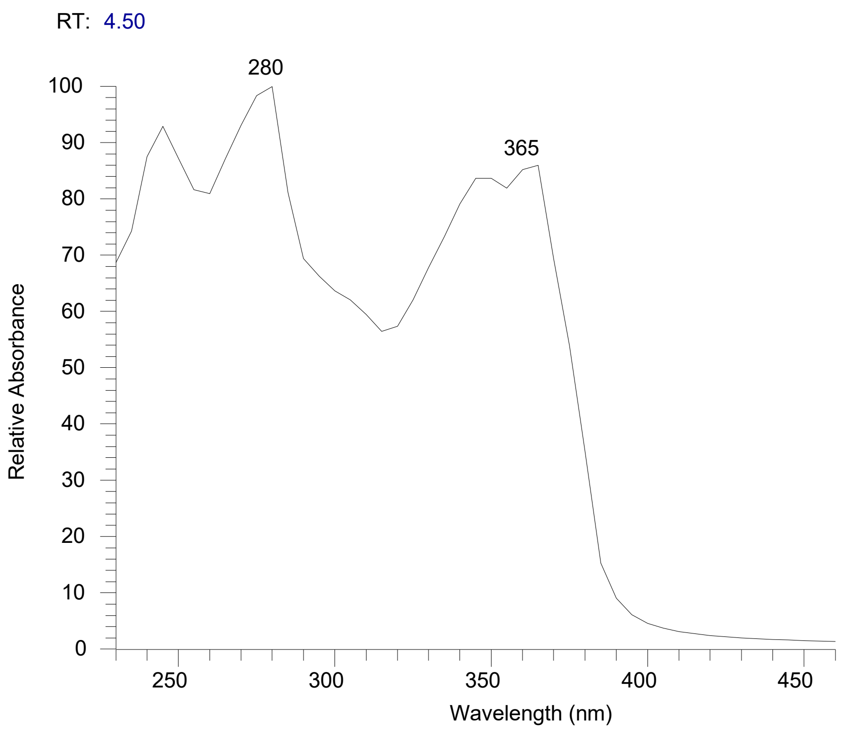


**Figure S1.** Mirror plots and UV absorbance spectrum supporting AOD and antibiotic Y mass feature annotations. Experimentally obtained spectrum is in black while *in silico* predicted spectral scores are in red. **A)** MS-FINDER $MS^2$ analysis of mass feature RT6.03_314.34134, annotated as 2-AOD-3-ol. **B**) MS-FINDER $MS^2$ analysis of mass feature RT4.48_319.04474, annotated as antibiotic Y. **C**) UV absorbance spectrum of associated with mass feature RT4.48_319.04474, annotated as antibiotic Y

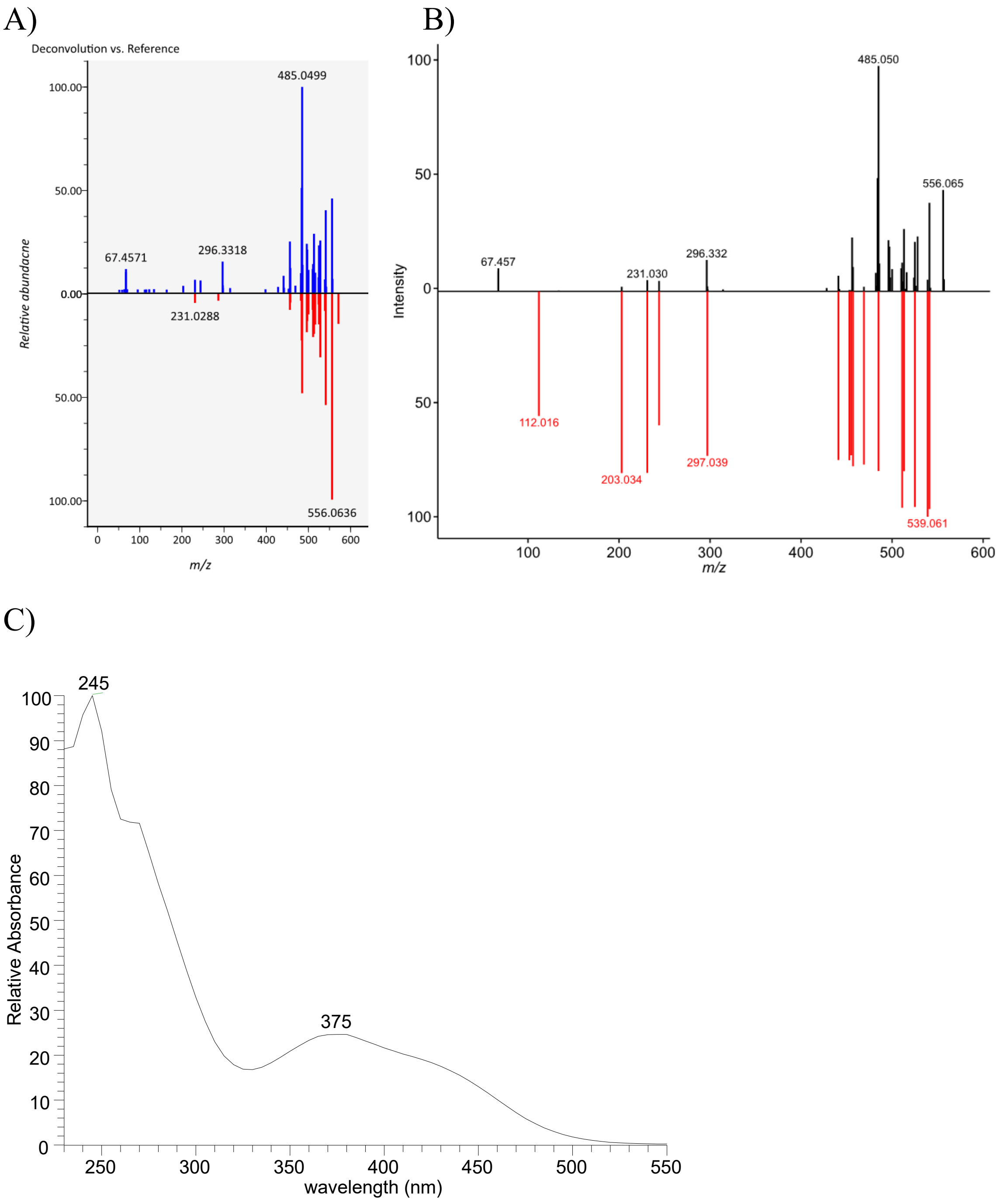


**Figure S2.** Mirror plots and UV absorbance spectrum supporting aurofusarin mass feature annotation. **A**) MS-DIAL mirror plot comparing aurofusarin-associated MS2 spectrum from MassBank to MS2 spectrum from mass feature RT4.95_571.08704, annotated as aurofusarin. Experimentally obtained spectrum is in blue (top plot), database matched $MS^2$ spectrum is in red (bottom plot). **B**) MS-FINDER $MS^2$ analysis of mass feature RT4.95_571.08704, annotated as aurofusarin. Experimentally obtained spectrum is in black while *in silico* predicted spectral scores are in red. **C**) UV absorbance spectrum of associated with mass feature RT4.95_571.08704, annotated as aurofusarin.

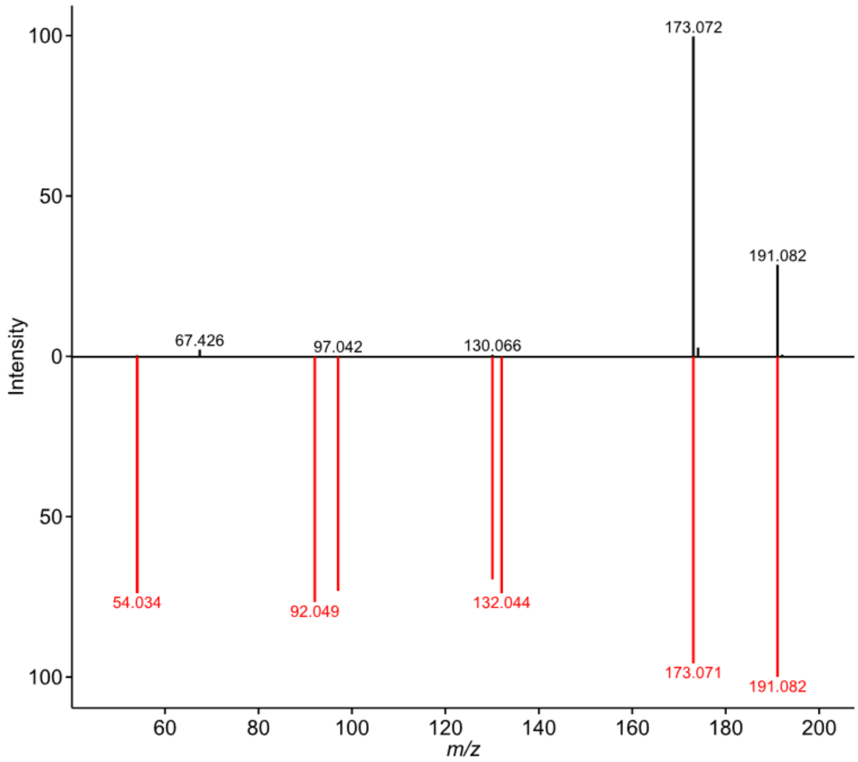


**Figure S3.** Mirror plots supporting chrysogine mass feature annotation. **A)** MS-FINDER $MS^2$ analysis of mass feature RT3.09_191.08173, annotated as chrysogine. Experimentally obtained spectrum is in black while *in silico* predicted spectral scores are in red.

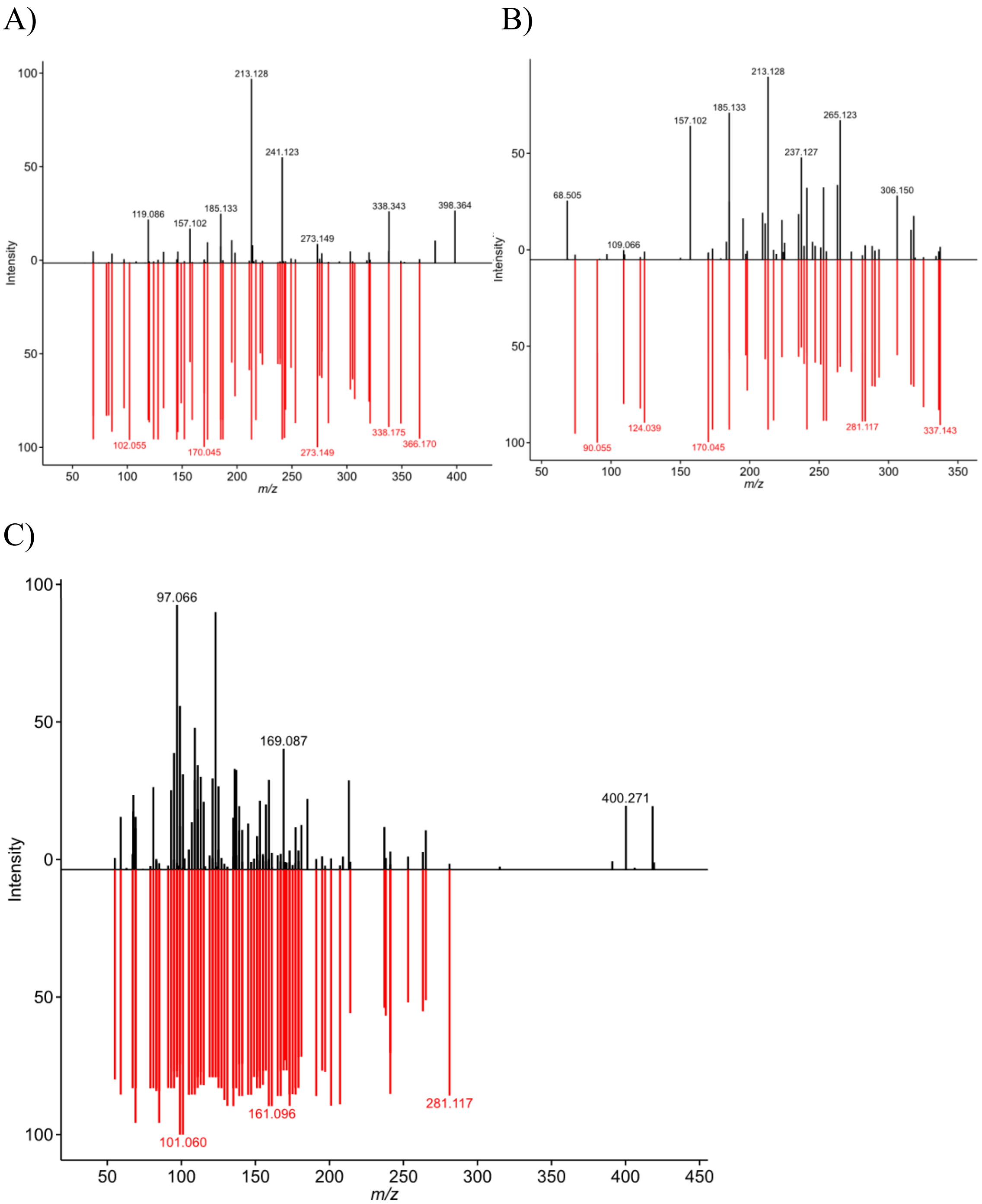


**Figure S4.** Mirror plots supporting fusarin mass feature annotations. Experimentally obtained spectrum is in black while *in silico* predicted spectral scores are in red. **A)** MS-FINDER MS$^2$ analysis of mass feature RT4.96_416.206545, annotated as fusarin A. **B**) MS-FINDER MS$^2$ analysis of mass feature RT4.61_432.20166, annotated as fusarin C. **C**) MS-FINDER MS$^2$ analysis of mass feature RT4.02_418.18588, annotated as fusarin PM.

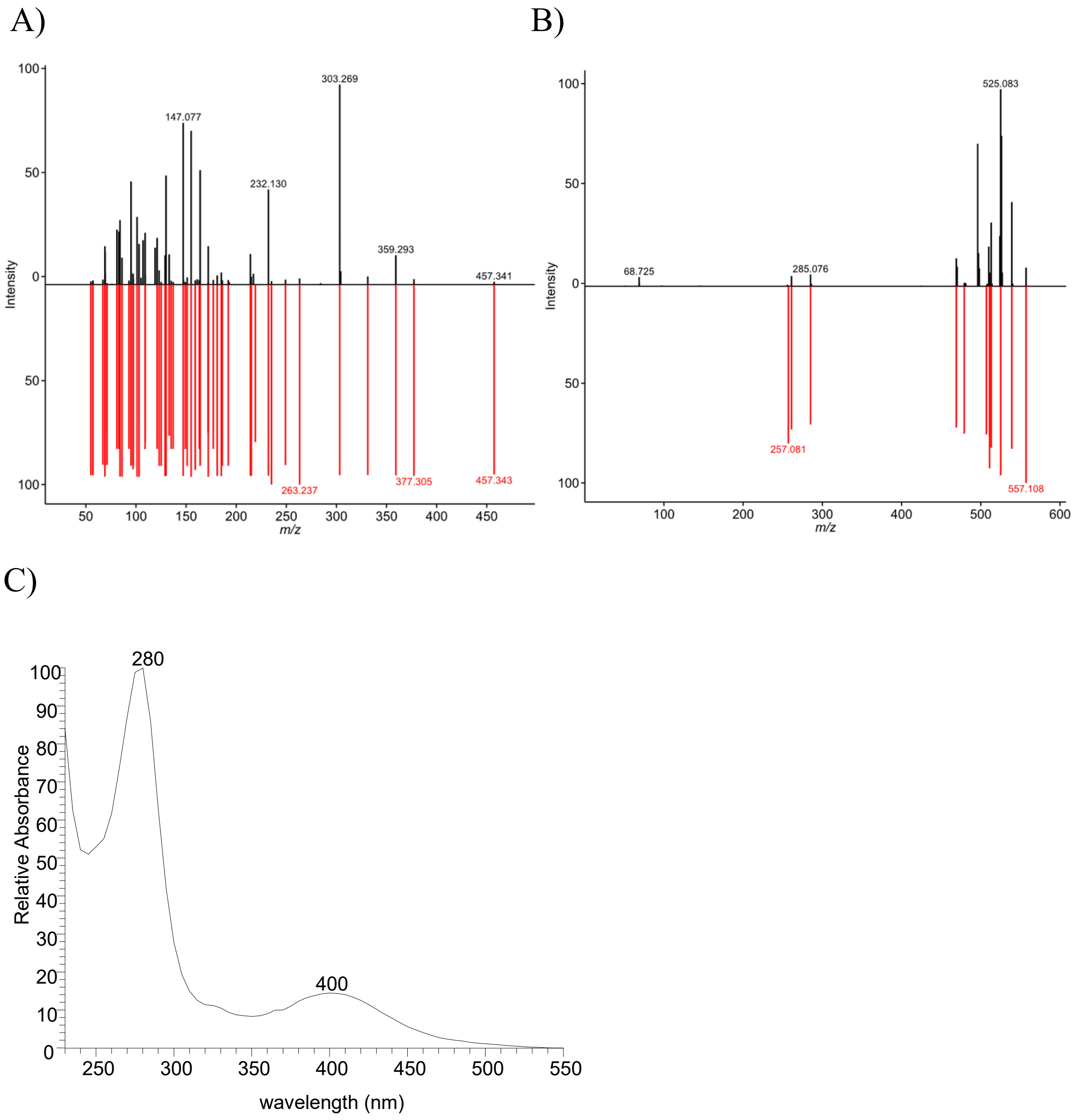


**Figure S5.** Mirror plot and UV absorbance spectrum supporting fusaristatin A and fuscofusarin mass feature annotations. Experimentally obtained spectrum is in black while *in silico* predicted spectral scores are in red. **A**) MS-FINDER MS$^2$ analysis of mass feature RT5.78_659.4387, annotated as fusaristatin A. **B**) MS-FINDER MS$^2$ analysis of mass feature RT5.38_557.10803, annotated as fuscofusarin. **C**) UV absorbance spectrum of associated with mass feature RT5.38_557.10803, annotated as fuscofusarin

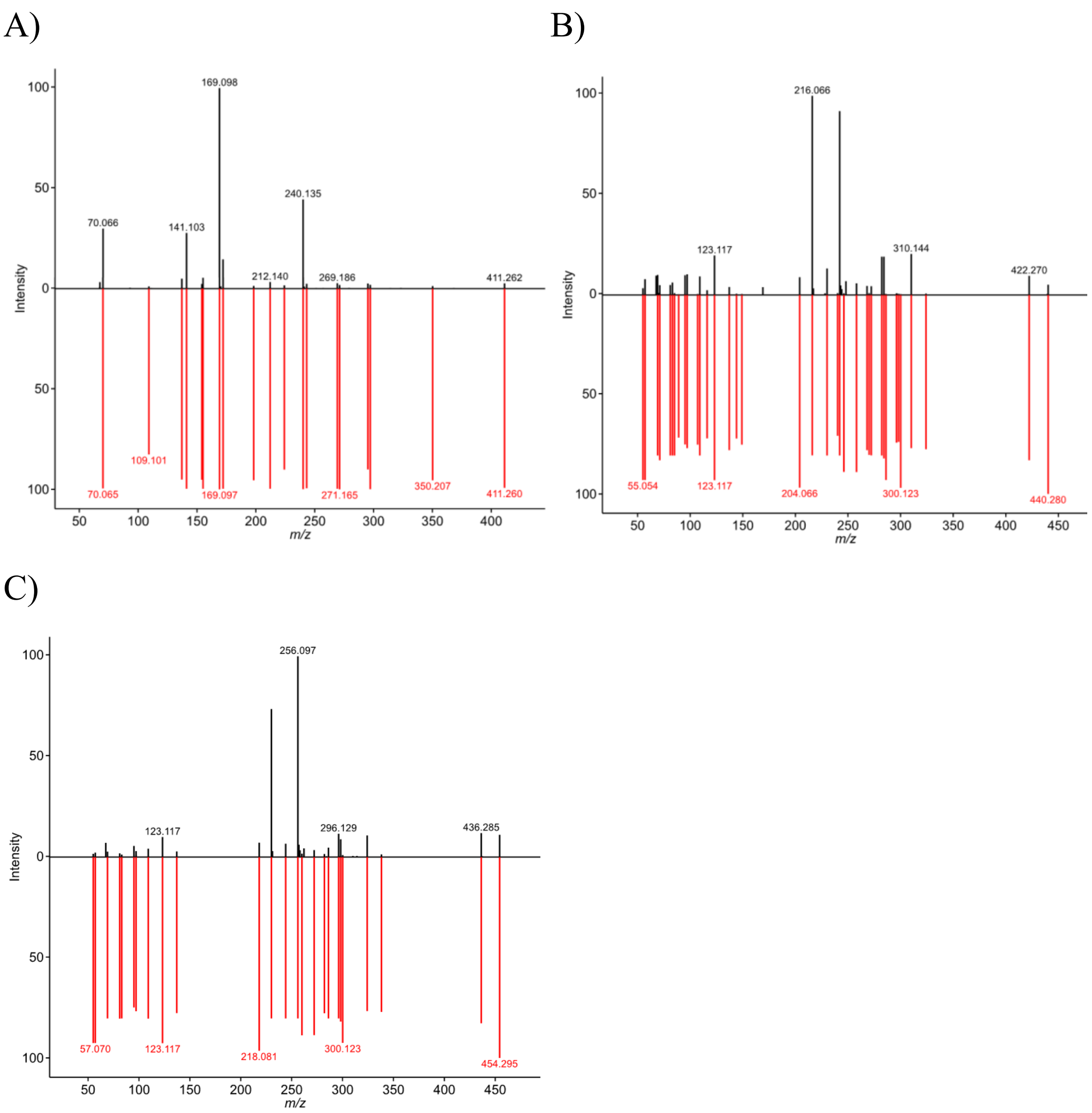


**Figure S6.** Mirror plots supporting JM-47 and sambutoxin mass feature annotations. Experimentally obtained spectrum is in black while *in silico* predicted spectral scores are in red. **A**) MS-FINDER $MS^2$ analysis of mass feature RT3.50_439.25485, annotated as JM-47. **B**) MS-FINDER $MS^2$ analysis of mass feature RT5.88_440.27936, annotated as N-desmethyl-sambutoxin. **C**) MS-FINDER $MS^2$ analysis of mass feature RT6.12_454.29507, annotated as sambutoxin.

A) B)

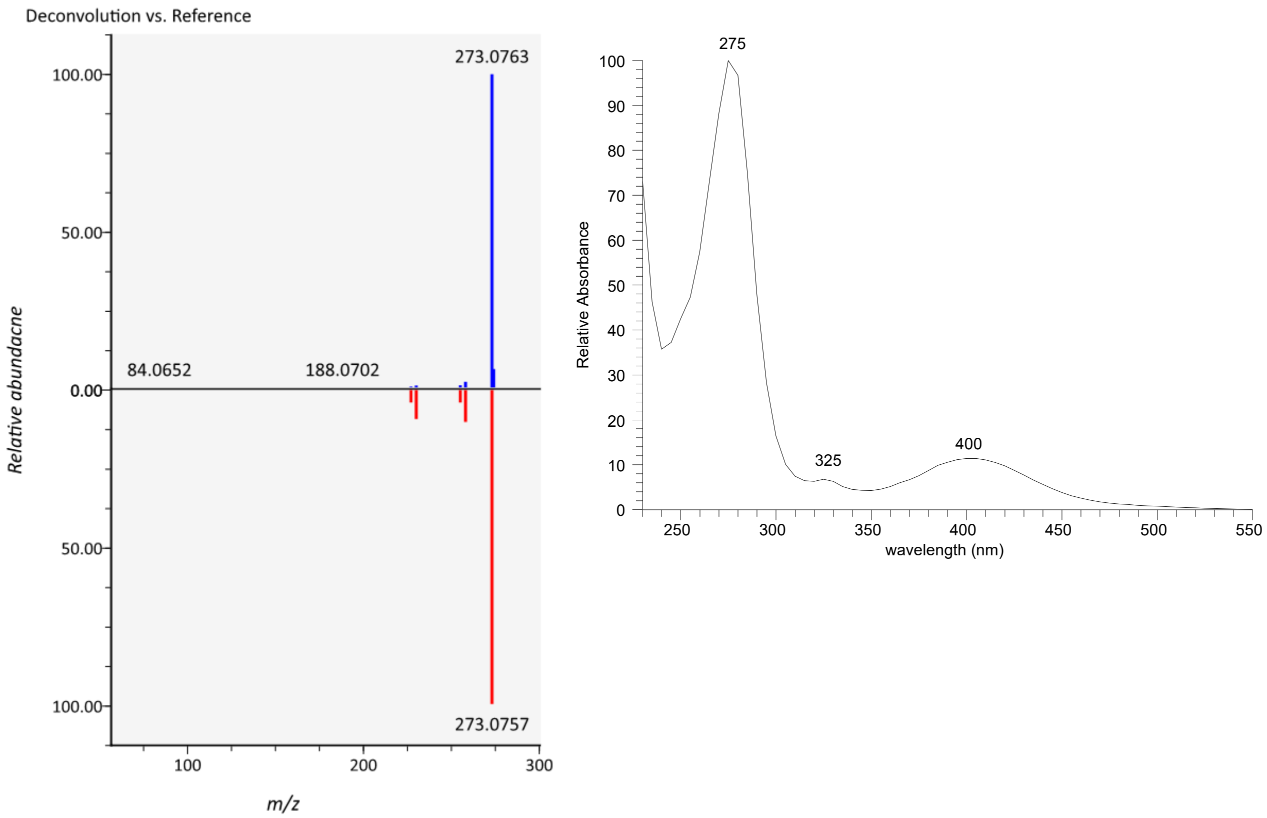


**Figure S7.** Mirror plot and UV absorbance spectrum supporting rubrofusarin mass feature annotation. **A**) MS-FINDER $MS^2$ analysis of mass feature RT5.33_273.07544, annotated as rubrofusarin. Experimentally obtained spectrum is in blue (top plot), database matched $MS^2$ spectrum is in red (bottom plot). **B**) UV absorbance spectrum of associated with mass feature RT5.33_273.07544, annotated as rubrofusarin.
